# When years of many different diagnosis may turn into one – a case of munchausen syndrome

**DOI:** 10.1192/j.eurpsy.2021.1733

**Published:** 2021-08-13

**Authors:** M.I. Lobo, A. Amorim, M.D.C. Cruz

**Affiliations:** Department Of Psychiatry 2, University Hospital Center of Algarve - Portimão Unit, Portimão, Portugal

**Keywords:** Munchausen syndrome, factitious disorder imposed on self

## Abstract

**Introduction:**

The term Munchausen syndrome was first used in 1951 after Baron von Münchhausen, a German nobleman known for his exaggerated storytelling. DSM-5 refers to it as factitious disorder imposed on self: “falsification of physical or psychological signs or symptoms, or induction of injury or disease, (…) in the absence of obvious external rewards”.

**Objectives:**

To report a case of Munchausen syndrome and highlight the impact on its physical and psychiatric approaches.

**Methods:**

Description of a clinical case based on medical records and a brief review on Munchausen syndrome.

**Results:**

A 57-year-old female, with no previous psychiatric history, was evaluated by Psychiatry for complaints of depression with suicidal ideation. She reported family conflicts and a list of medical conditions and surgical interventions. According to the patient she was waiting for a cardiac transplant and said she had type 1 diabetes, myasthenia gravis, hepatic steatoses, dyslipidemia, hyperuricemia, mitral valve prolapse and was submitted to a thymectomy and cervical herniated disc surgery. She was on many different prescription pills. Even though she had blocked the access to her clinical records in other institutions, at our hospital she had multiple admissions to the emergency room, numerous follow-up appointments of different specialties and several allergies documented. She displayed many incoherencies throughout the interview, had a circumstantial speech and exuberant appearance.

**Conclusions:**

Munchausen syndrome remains a challenging diagnosis to physicians. This condition is not only associated with significant morbidity and mortality, but also with unnecessary tests and procedures, iatrogenesis, prolonged hospitalizations and increased health costs.

**Disclosure:**

No significant relationships.

